# Fast convergence of learning requires plasticity between inferior olive and deep cerebellar nuclei in a manipulation task: a closed-loop robotic simulation

**DOI:** 10.3389/fncom.2014.00097

**Published:** 2014-08-15

**Authors:** Niceto R. Luque, Jesús A. Garrido, Richard R. Carrillo, Egidio D'Angelo, Eduardo Ros

**Affiliations:** ^1^Department of Computer Architecture and Technology, University of Granada (CITIC)Granada, Spain; ^2^Consorzio Interuniversitario per le Scienze Fisiche della Materia (CNISM)Pavia, Italy; ^3^Neurophysiology Unit, Department of Brain and Behavioral Sciences, University of PaviaPavia, Italy; ^4^Brain Connectivity Center, C. Mondino National Neurological InstitutePavia, Italy

**Keywords:** cerebellar nuclei, inferior olive, long-term synaptic plasticity, learning consolidation, modeling

## Abstract

The cerebellum is known to play a critical role in learning relevant patterns of activity for adaptive motor control, but the underlying network mechanisms are only partly understood. The classical long-term synaptic plasticity between parallel fibers (PFs) and Purkinje cells (PCs), which is driven by the inferior olive (IO), can only account for limited aspects of learning. Recently, the role of additional forms of plasticity in the granular layer, molecular layer and deep cerebellar nuclei (DCN) has been considered. In particular, learning at DCN synapses allows for generalization, but convergence to a stable state requires hundreds of repetitions. In this paper we have explored the putative role of the IO-DCN connection by endowing it with adaptable weights and exploring its implications in a closed-loop robotic manipulation task. Our results show that IO-DCN plasticity accelerates convergence of learning by up to two orders of magnitude without conflicting with the generalization properties conferred by DCN plasticity. Thus, this model suggests that multiple distributed learning mechanisms provide a key for explaining the complex properties of procedural learning and open up new experimental questions for synaptic plasticity in the cerebellar network.

## Introduction

Biological motor learning is characterized by several attributes: it usually proceeds through a rapid convergence toward a stable state, it can become consolidated into persistent memory, it can be generalized to analogous cases, it can proceed along multiple consolidation steps, and it can be saved. The cerebellum is known to play a critical role in learning relevant patterns of activity for adaptive motor control, but the underlying network mechanisms are only partly understood.

The cerebellum is also widely assumed to act as a control module which is embedded in a feedforward control loop (Goodwin and Sin, 1984; Ito, 1984; Miall et al., 1993; Wolpert and Miall, 1996; Turrigiano and Nelson, 2004) capable of evaluating both the incoming sensory information from the environment and the information provided by the system itself (propioception) before the motor control action is sent to the body plant. This means that the cerebellar controller manages the sensory information to deliver the best motor commands to accomplish the desired movement.

### Cerebellar motor-control-loop considerations

A pure feedforward control system is able to deliver the precise set of motor commands for the body-plant and make corrections during the movement without continuously checking the motor output (Schweighofer et al., 1998). Conversely, a system equipped with an adaptable forward controller exploits a previous trial-and-error learning process in order to later recognize all possible sensorial states that may be encountered and accordingly deliver on-time efficient corrective terms. In a real manipulation task, the environmental conditions are continuously changing and the forward controller continuously tunes motor commands to cope with these changing environmental conditions (Bastian, 2006).

**Figure 1:**
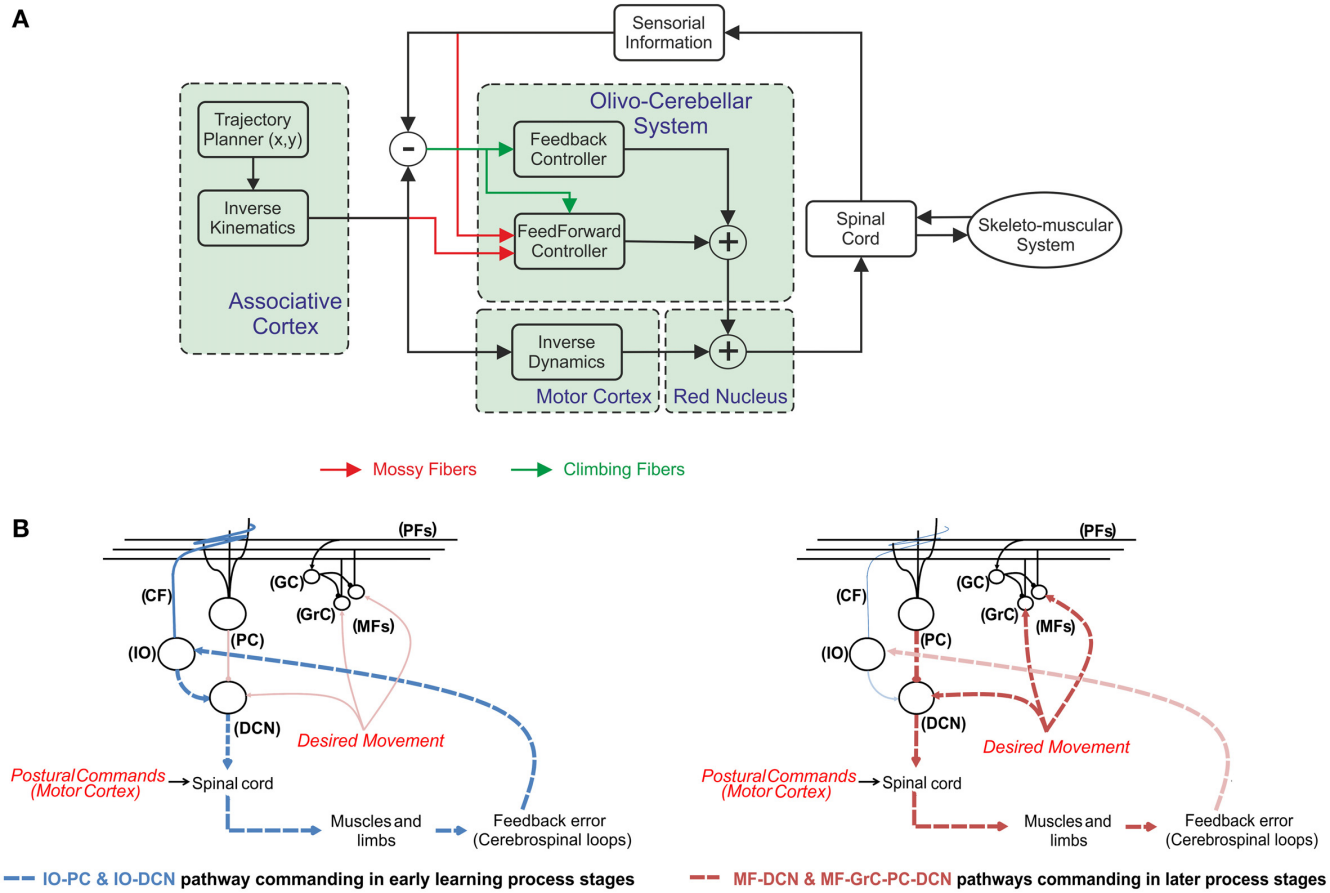
**The cerebellum operating in a feedforward control system. (A)** The mossy fibers are thought to provide information referring to the desired plant motor output from motor cortex and the current sensory information referring to the actual state of the body parts (i.e., joint positions/velocities of the upper-limbs of the body-plant). According to the Marr–Albus model (Marr, 1969; Albus, 1971) the climbing fibers are assumed to carry error-related information when moving, thus providing a teaching signal to the cerebellum. By using this error-based-teaching signal the cerebellum is able to learn the corrective actions in a trial-and-error process. When the cerebellar model is not able to deliver add-on torque terms to compensate deviations in the system (for instance during the early learning stages) the general rule consists of adding a feedback to stabilize the open-loop system. **(B)** Different control pathways during the learning process. The relevant information flow is represented by dashed lines in each learning stage. A fast response gain control is delivered by IO-DCN connection, thus supplying stability in early learning-process stages (dashed blue lines). In later learning-process stages the two control pathways (dashed red lines); the internal MF-GrC-PC-DCN and the more external MF-DCN command the control action. Whilst IO-DCN action decays throughout the learning process its control action is assumed and improved by these two long-term adaptive pathways.

According to this scheme (Figure 1A), the cerebellum operates as a forward controller for the motor commands generated in the motor cortex. The motor cortex generates a crude inverse model of the skeleton-muscular system. The cerebellum is able to learn, refine, and store accurate models of the skeleton-muscular system providing both the precise timing of agonist-antagonist muscle pairs and the force and stiffness control (Van Der Smagt, 2000). Obviously, the precise timing and force of muscles in a manipulation task depend on the object to be handled (more precisely, on the dynamic model of the object under manipulation; Turrigiano and Nelson, 2004).The cerebellar model must translate the actual/desired plant commands (in joint coordinate space) to corrective/prior motor values (in torque control actions). These latter corrective commands have to be fed into the body-plant along with the crude inverse model terms. This indeed represents a convenient solution, since several different corrections could easily be accomplished by the adaptable forward controller, whereas the possibility of switching/interpolating between different inverse models to deal with this changeable environment features (Wolpert and Kawato, 1998; Haruno et al., 1999, 2001; Petkos et al., 2006; Chai et al., 2008) would demand an overwhelming storage capability.

Whilst the cerebellum can indeed compensate mismatches in the internal models, its trial-error learning process may require a long time. This also means that, before the learning process is completed, the motor system works in an open-loop and any perturbation could easily destabilize the body-plant control scheme. This undesirable situation has been traditionally circumvented by adding a sensory feedback control loop accompanied on most occasions with some kind of feedback controller (Kawato and Gomi, 1992; Stroeve, 1997; Desmurget and Grafton, 2000; Kalveram et al., 2005). This latter ranges from a simple proportional—derivative control (Van Der Smagt, 2000), which ensures stability (Arimoto, 1984), to other more sophisticated controllers following the general structure shown in Figure 1A.

### The olivary-deep cerebellar nuclei system

An important advancement was brought in by the discovery of long-term synaptic plasticity between parallel fibers (PFs), and Purkinje cells (PCs), which drives learning under inferior olive (IO) action, thus regulating the background activity of PCs and supplying a teaching signal (Bazzigaluppi et al., 2012; De Gruijl et al., 2012) to the cerebellum. Although this mechanism supports the core process of error-correction in the motor learning theory and subsequent derivations, this same mechanism alone can only account for limited aspects of biological learning. A new spectrum of possibilities was opened by the discovery on multiple forms of synaptic plasticity (Hansel et al., 2001; Evans, 2007; Ohtsuki et al., 2009; D'Angelo, 2011; Gao et al., 2012). A recent model has shown that plasticity at deep-cerebellar nuclei (DCN) synapses can indeed account for learning on multiple time scales and for generalization (Garrido et al., 2013a). Moreover, corrective torque values are better determined by introducing granular layer plasticity (Schweighofer et al., 2001). Detailed analysis of the granular layer network has revealed that specific combinations of plasticity at the different synapses can change the output pattern on the millisecond time-scale (Garrido et al., 2013b). Moreover, modeling of long-term synaptic plasticity at DCN synapses has allowed the explanation of the double learning time-scale characterizing the cerebellum (Medina and Mauk, 1999, 2000). Nevertheless, in all these cases, cerebellar learning demands hundreds of repetitions, suggesting that additional mechanisms are required to speed-up convergence. This problem has been circumvented by most functional cerebellar approximations by means of combining the fundamental idea of a negative feedback control able to handle explicit peripheral sensorial measurement with the notion of some kind of adaptive inverse control (Kalveram et al., 2005). In addition, the coexistence of different forms of plasticity based on local activity levels require some kind of stabilizing mechanisms operating in the local neural circuit to prevent instability as a result of over-excitation or saturation (Turrigiano and Nelson, 2004). Therefore, there must be some biological mechanism providing speed-up and stabilization of learning.

In this article we have considered the putative role of the IO-DCN connection by endowing it with adaptable weights and exploring its implications in a closed-loop robotic manipulation task. DCN neurons are innervated by excitatory synapses from climbing fiber collaterals (CFs) and MFs as well as by inhibitory synapses from PCs. The effect that these excitatory and inhibitory connections produce still remains uncertain (Bengtsson and Hesslow, 2006; Uusisaari and De Schutter, 2011). Our results show that IO-DCN plasticity accelerates convergence of learning by up to two orders of magnitude without conflicting with the generalization properties conferred by MF-DCN and PC-DCN plasticity. By means of Hebbian rules, the IO-DCN connection can adjust its synaptic weight and the excitability of DCN neurons provides a *built-in feed-back controller* generating fast corrections at early stages during learning. Thus, this model implies that multiple distributed learning mechanisms may provide the key for explaining the complex properties of biological learning and prompts the search for yet undetermined forms of synaptic plasticity in the cerebellar network.

## Methods

This section describes the working principles of the proposed mechanistic cerebellar model as well as the existing relationship between the functionality of the cerebellar system and its underlying layer structure. The section is divided into three main conceptual blocks; starting with the description of both the cerebellar topology and the implemented plasticity mechanisms; continuing with the cerebellar control loop description and finishing with the benchmark trajectory used for quantitative evaluation experiments.

### Cerebellar model

In order to develop our cerebellar model, we adopted a functional scheme in which the effort was focused on maintaining the functional information processing features of the cerebellar micro-circuitry but using cells with analog activity values (instead of an explicit spiking representation) (Ostojic and Brunel, 2011). To this aim, each cerebellar layer was implemented as a set of values representing the firing rate of each cell, thus allowing the mathematical study of the functional role that the IO-DCN connection may have in both cerebellar motor learning and control. The proposed cerebellar model took inspiration from the biological cerebellar micro-complex circuit. It uses several forms of plasticity mechanisms at several cerebellar sites which work in balance with the IO-DCN connection acting as control signal over the cerebellar output in a neurobotics control scenario.

Our model consists of four main layers:

- Granular layer: a state-generator model following Yamazaki and Tanaka's hypothesis has been implemented. The granular layer acts as an internal clock generating different time stamps along the executed trajectory (Yamazaki and Tanaka, 2005; Honda et al., 2011) (time stamps which are repeated in each trial). The current time along the arm-plant trajectory trial is unambiguously represented by using 500 different input states. These 500 input states are the result of the division of the arm trajectory duration (1 s) by the simulation time step (2 ms). Adopting a sparse representation, these states emulate 500 PFs sequentially activated.
- Purkinje-cell layer: The activity at PCs is defined in Equation (1):
  (1) $$\begin{array}{l} PC_{i}(t)=f_{i}\left(PF(t)\right),\\ \;where\;i\in \left\{1,2,\ldots ,Number\;of\;muscles\right\} \end{array}$$
  where *PC_i_*(*t*) represents the average firing rate of the PCs associated to the *i*th muscle. Our robot arm-plant presents 3 agonist-antagonist pairs of muscles, representing a total of 6 muscles. *f*_i_ defines the function which matches each granular layer state (active PF) with a particular output firing rate at each PC. This function is modified during the learning process of a particular movement. In this model the output activity at different cell layers (PCs, MFs, and CFs) has been normalized between 0 (representing the absence of activity) and 1 (representing the maximum firing rate of the cell layer).
- Mossy fibers: Our cerebellar model assumes that mossy fibers (MFs) transmit a baseline neural activity during the trajectory execution according to studies of eye blink conditioning experiments (Yamazaki and Tanaka, 2007a,b, 2009).
- DCN cells: The activity of these nuclei cells has traditionally been related with both the excitatory-activity integration coming from MFs and the inhibitory-activity integration from PCs, neglecting the impact of IO-DCN connections. Due to the low number of MFs and climbing fibers (CFs) in comparison to granule cells (GrCs), the capacity of these fibers for generating a sparse representation of different cerebellar states seems to be very limited (in fact, in our model MFs can be understood more as a baseline global activity/term provider). This fact shows that the reported synaptic plasticity at MF-DCN synapses (Racine et al., 1986; Medina and Mauk, 1999; Pugh and Raman, 2006; Zhang and Linden, 2006) in balance with the IO-DCN connection activity can induce the adjustment of gain control through plasticity at DCN synapses. The Equation (2) describes the DCN cell behavior:
  (2) $$\begin{array}{l} DCN_{i}(t)\;=\;W_{MF\;-\;DCN,i}\;-\;PC_{i}(t)\cdot \\ \;W_{PC\;-\;DCN,i}\;+\;IO_{i}(t)\;\cdot \;W_{IO\;-\;DCN,i},\\ where\;i\;\in \;\left\{1,2,\ldots ,Number\;of\;muscles\right\} \end{array}$$
  *DCN_i_*(*t*) represents the average firing rate of the DCN cells associated to the *i*th muscular group and *W*_*MF* − *DCN*,*i*_ is the synaptic strength of the MF-DCN connection at the muscular group *i*, *W*_*PC* − *DCNi*_ represents the synaptic strength of the PC-DCN connection of the *i*th muscle. Finally *IO_i_*(*t*) represents the average firing rate of the CFs associated to the *i*th muscle where *W*_*IO* − *DCNi*_ represents the synaptic strength of the IO-DCN connection of the *i*th muscle.

All these synaptic strengths are progressively adapted during the learning process according to different synaptic plasticity mechanisms which will be explained in detail in the following section.

### Synaptic plasticity

The cerebellum model was endowed with multiple forms synaptic plasticity, which can be summarized in the following equations.

#### PF-PC, MF-DCN, and PC-DCN long-term synaptic plasticity

Following on from our previous article (Garrido et al., 2013a), the present model implements different forms of synaptic plasticity as follows:

PF-PC synaptic plasticity:

(3) $$\begin{array}{l} \Delta W_{PF_{j}\;-\;PC_{i}}(t)\;=\{\begin{array}{c} \frac{LTP_{Max}}{{\left(IO_{i}(t)+1\right)}^{\alpha }}-LTD_{Max}\cdot IO_{i}(t),\\ \;if\;PF_{j}\;is\;active\;at\;t\\ 0\;otherwise \end{array}\\ where\;i\in \left\{1,2,\ldots ,Number\;of\;muscles\right\} \end{array}$$

where Δ*W_PF_j_ − PC_i__*(*t*) represents the weight change between the *j*th PF and the target PC associated with the *i*th muscle. *IO_i_* (*t*) stands for the current activity coming from the associated climbing fiber (which represents the normalized error along the executed arm plant movement), *LTP_Max_* and *LTD_Max_* are the maximum long term potentiation/long terms depression (LTP/LTD) values, and α is the LTP decaying factor. In the experiments α is set to 1000 in order to ensure a fast LTP action decreasing (Garrido et al., 2013a).

MF-DCN synaptic plasticity:

(4) $$\begin{array}{l} \Delta W_{MF\;-\;DCN_{i}}(t)\;=\frac{LTP_{Max}}{{\left(PC_{i}(t)+1\right)}^{\alpha }}-LTD_{Max}\cdot PC_{i}(t),\\ where\;i\in \left\{1,2,\ldots ,Number\;of\;muscles\right\} \end{array}$$

where Δ*W*_*MF* − *DCNi*_(*t*) represents the weight change between the active MF and the target DCN associated with the *i*th muscle, *PC_i_*(*t*) is the current activity coming from the associated PCs, *LTP_Max_*, and *LTD_Max_* are the maximum LTP/LTD values, and α is the LTP decaying factor; α is set to 1000 in order to ensure a fast LTP action decreasing (Garrido et al., 2013a).

PC-DCN synaptic plasticity:

(5) $$\begin{array}{l} \Delta W_{PC_{i}\;-\;DCN_{i}}(t)=LTP_{Max}\cdot PC_{i}{\left(t\right)}^{\alpha }\cdot \\ \;\left(1-\frac{1}{{\left(DCN_{i}(t)+1\right)}^{\alpha }}\right)-LTD_{Max}\\ \;\left(1-PC_{i}(t)\right),\\ where\;i\in \left\{1,2,\ldots ,Number\;of\;muscles\right\} \end{array}$$

where Δ*W*_*PCi* − *DCNi*_(*t*) is the synaptic weight adjustment at the PC-DCN connection reaching the DCN cell associated with the *i*th muscle, *PC_i_*(*t*) is the current activity coming from the associated PCs and finally DCN is the current activity regarding DCN cells present. Again, α is set to 1000 in order to ensure a fast LTP action decreasing (Garrido et al., 2013a).

For these synapses, considerations and parameterizations are identical to those reported previously (Garrido et al., 2013a) and are not repeated here.

#### IO-DCN synaptic plasticity

The MF-DCN synaptic plasticity mechanism was previously hypothesized to be a proper cerebellar gain controller which self-adapts its maximum output activity to minimize the inhibition impact of the inhibitory pathway already described (Garrido et al., 2013a). Nevertheless, this cerebellar gain controller reaches the adequate state through the learning process. This involves a time period in which the control action is not delivered properly which makes the system prone to become unstable. The cerebellum, during this learning process, is able to supply enough control action to avoid these possible destabilization inconveniences. Furthermore, the feedback action in cerebellar motor control is well accepted (Kawato and Gomi, 1992; Stroeve, 1997; Desmurget and Grafton, 2000; Kalveram et al., 2005) and neurophysiologic evidence also exists suggesting that the primary motor cortex is involved in this feedback loop (Sergio et al., 2005). Concretely, there is a dense projection from primary motor cortex to the spinal cord, often directly onto motor neurons, and correlations between primary motor cortex activity and end-effector kinematics (Todorov, 2000). Hence, proprioceptive signals encoding for instance position error information (inputs) are put in relation with the corrective cerebellar output, thus leading us to believe that the IO-DCN connection might implement this loop.

According to Figure 1B, DCN input signals (proprioceptive signals) are received from two differentiated pathways. The first pathway reaches the DCN cells through the cerebellar cortex. This feedback system has been widely hypothesized to be the main adaptive pathway in which cerebellar learning takes place (Strata, 2009). The second pathway reaches DCN directly by means of the CF collaterals and MFs. The input information (proprioceptive signals encoding, for instance, position errors) is then related with the corrective cerebellar output. Whilst MF activity arriving to the DCN may work as a non-specific baseline, which is modulated by the PC specific inhibition pathway; the role of the second excitatory pathway consisting of CF collaterals is unclear, but its location allows it to work as a feedback controller as shown in Figure 1B. Our working hypothesis is based on a fast response gain action delivered by IO-DCN connection, thus supplying stability.

At the very beginning of any manipulation task, if the hand carries an unknown object affecting the arm dynamics/kinematics the inverse model does not match with the real plant and the learning process starts acquiring the manipulated object model. After some time (through the learning process) this IO-DCN action decays and its control action is assumed and improved by the two pathways whose actions have been previously described; the internal MF-GrC-PC-DCN and MF-DCN. The assumption involving a relevant action of this connection (IO-DCN) at early stages of the learning process implies that the initial synaptic strength set by this learning law must be in the same range as the one assumed in subsequent learning stages by the other two control pathways (these pathways are illustrated in Figure 1B).

According to Ito (2008) this possible feedback controller must generate a command in motor cortex capable of tuning the viscoelastic properties of musculoskeletal system (tension-length and tension-velocity relation). This assumption can be seen as a fast and short adaptation of the cerebellar circuitry (synaptic weight strength) to cope with this required initial control action. Within our working hypothesis, the plasticity mechanism was implemented to range adequately the initial synaptic strength of DCN cells driven by the IO as defined by Equation (6).

(6) $$\begin{array}{l} \Delta W_{IO\;-\;DCN,i}(t)=MTP_{Max}\cdot IO_{i}(t)-\frac{MTD_{Max}}{{\left(IO_{i}(t)+1\right)}^{\alpha }}\\ where\;i\;\in \;\left\{1,2,\ldots ,Number\;of\;muscles\right\} \end{array}$$

where Δ*W*_*IO* − *DCN,i*_(*t*) represents the differential synaptic weight factor related to the active connection at time *t* [whose associated activity state corresponds to *IO_i_*(*t*)]. The connection corresponds to the DCN cell associated to the *i*th muscle. This weight can be seen as a fast modulation adaptation term, *MTP_Max_* and *MTD_Max_* (modulating term plasticity) are both the maximum MTP/MTD values to be applied at any time. Both terms present an enormous value in comparison to LTP/LTD values previously described, thus ensuring a fast response and a negligible contribution to the learning process in the long term. *IO_i_* is the normalized current activity in the range [0, 1] coming from the associated climbing fiber (which represents the current error translated into a control signal along the executed arm-plant movement), and finally α defines the MTD decaying factor (set to 1000 in our simulations ensuring a fast MTD action decreasing).

For instance, in a scenario with a significant mismatch between the inverse model and the robot-arm plant, there is a high activity at IO (ranged in [0, 1]), which means a high ongoing error value. In this situation, the potentiation term dominates the expression, the incremental difference Δ*W* to be applied is high and in a very few time-steps this weighting factor is adapted. Although from the beginning the action of the other long-term synaptic mechanisms is active, it still requires some time to arrive at stable weights (due to its slower dynamics). When the error is low, the activity of the IO is closed to 0 and the depression term dominates, which means that the weight factor is quickly decreased. As we can see the potentiation/depression action compensates each other. What it is quickly learnt due to an action is quickly forgotten due to the opposite action.

In order to obtain a numerical evaluation of the modulated term impact in the convergence speed process (**Figure 6**), the normalized mean absolute error (MAE) convergence speed defined in Luque et al. (2011b) has been used. This measurement is defined as the number of needed samples (iterations of the movement) to reach the final error average. To normalize the measurement, the cerebellar configuration without IO-DCN corrective action was conceived to be the worst possible scenario, thus assigning a value of 1 to the obtained number of samples needed to reach the final error average in the absence of IO-DCN terms (i.e., the slowest possible convergence speed).

### Cerebellar control loop

**Figure 2:**
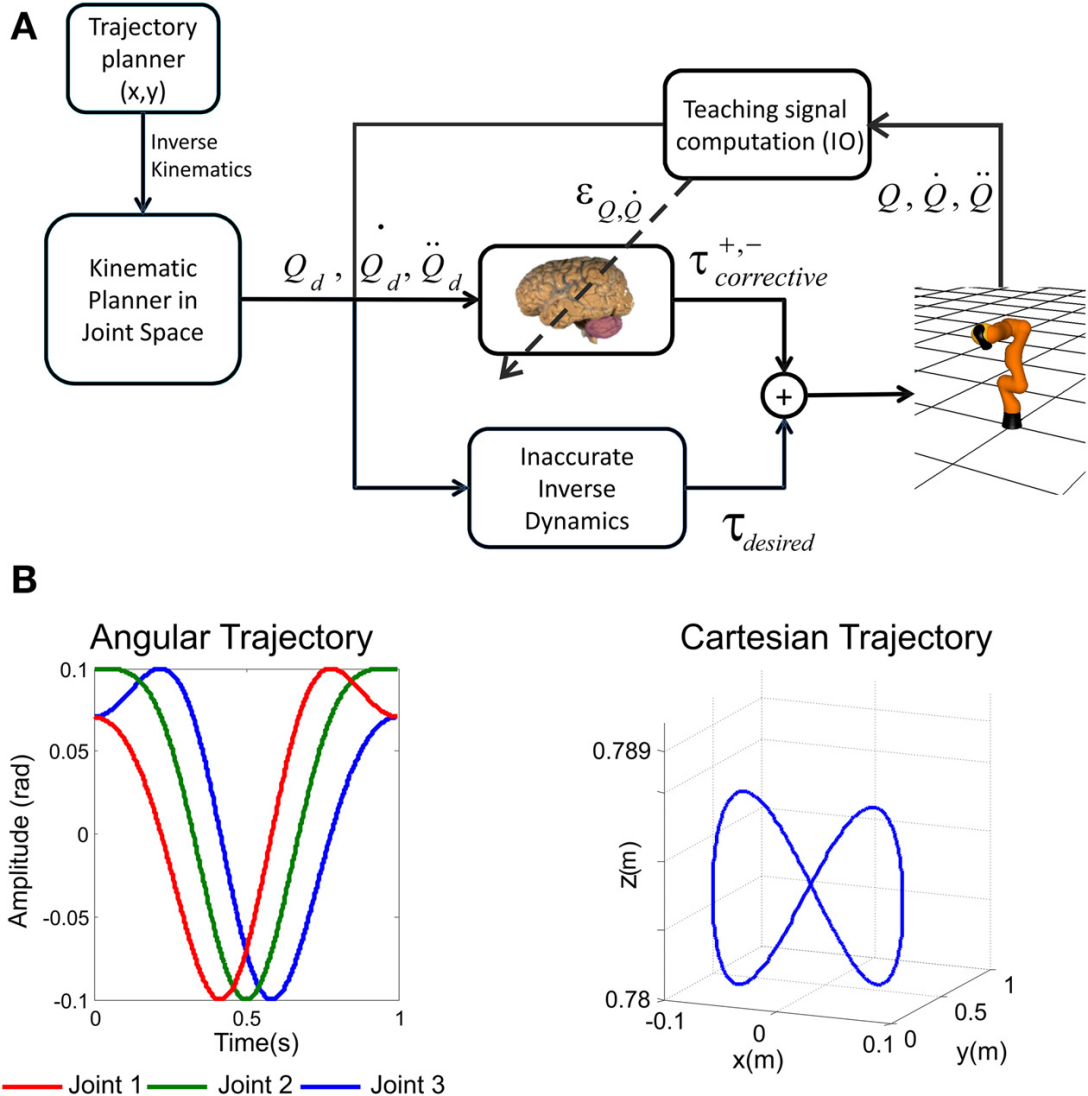
**Cerebellar control loop and benchmark trajectory. (A)** The adaptive cerebellar module delivers corrective torque values (τ_corrective_) to compensate for deviations in the crude inverse dynamic module when manipulating an object of significant weight. In this feedforward control loop, the cerebellum receives a teaching error-dependent signal and the desired arm state (*Q_d_*, ${\dot{Q}}_{d}$, ${\ddot{Q}}_{d}$) so as to produce the adaptive corrective actions. **(B)** Three-joint periodic benchmark trajectory suitable for testing the kinematic and dynamical properties of the robot arm and the application area. Fast movements in a smooth pursuit task composed of vertical and horizontal sinusoidal components are able to reveal the whole robot arm dynamic properties (Hoffmann and Petkos, 2007). The left panel represents angular coordinate per joint followed by the light weight robot, the right panel plots the robot end-effector trajectory in euclidean space.

As we have pointed out, the adopted control loop is based on a feedforward scheme in conjunction with a crude inverse dynamic model of the arm plant (Figure 2A). This feedforward action is complemented with the presented cerebellar model which acts as a feedback controller in part due to the IO-DCN connection. In our model, an inverse kinematic module translates the desired trajectory into arm-joint coordinates. Another module (inverse dynamics) based on a recursive Newton-Euler algorithm generates crude step-by-step motor commands corresponding to the desired trajectory.

Some studies suggest that the central nervous system may, in fact, plan and execute voluntary movements in a sequential process. The brain would first plan the optimal trajectory in task-space coordinates, then translate them into intrinsic-body coordinates, and then finally, generate the appropriate motor commands to achieve theses transitions (Houk et al., 1996; Nakano et al., 1999; Todorov, 2004; Hwang and Shadmehr, 2005; Izawa et al., 2012; Passot et al., 2013). According to these studies, the association cortex provides the desired trajectory in body coordinates and conveys them to the motor cortex which, in turn, calculates the motor commands by using an inverse dynamic arm model. On the one hand, the spinocerebellum-magnocellular red nucleus system provides an internal neural accurate model of the musculoskeletal dynamics of the system which is learned with practice by sensing the result of movements (Kawato et al., 1987). Conversely, the cerebrocerebellum-parvocellular red nucleus system is thought to provide such a crude internal neural model of the inverse-dynamics of the musculoskeletal system (Kawato et al., 1987). The crude inverse dynamic model works in conjunction with the dynamical model (given by the spinocerebellum-magnocellular red nucleus system) to update the motor commands according to a possible predictable error when executing a movement. As illustrated in Figure 1A, the cerebellar pathways follow a feedforward scheme, in which only information about sensorial consequences of non-accurate commands is available.

### Benchmark trajectory

We have integrated a simulated light-weight robot (LWR) arm within our feedforward control loop. The simulated-robot-plant physical characteristics can be dynamically modified to match different contexts (in our experiments different contexts mean that the object manipulated by the robot, payload, has different weights). This LWR (Hirzinger et al., 2000; Albu-Schäffer et al., 2007) robot is a 7-DOF arm composed of revolute joints. In our experiments we used the first (we will refer to it as Q1), second (Q2), and fifth joint (which we label as Q3), maintaining the others fixed.

Our aim was to select a benchmark trajectory which reveals the dynamic properties of our robot plant arm. The robot dynamics have been fully considered as indicated in Supplementary Material. The choice of a trajectory to test our cerebellar control relies on the kinematic and dynamical properties of the robot arm and the application area. We have chosen fast movements in a smooth pursuit task composed of vertical and horizontal sinusoidal components (Kettner et al., 1997; Van Der Smagt, 2000) (1 s for the whole target trajectory) to study how inertial components (when manipulating objects) are inferred by the cerebellar module (Luque et al., 2011a,c). Slow movements would hide changes in the dynamics of the arm+object model when manipulating objects of different weights since they would have negligible impact. The target trajectory describes an “8-shape” defined by Equation (7) in angular coordinates.

(7) $$\begin{array}{l} Q_{n}\;=\;A_{n}\cdot \sin\left(\left(-4\pi t^{3}+6\pi t^{2}\right)+C_{n}\right)\\ where\;n\;=\;\left\{1,\ldots ,number\;of\;links\right\} \end{array}$$

where *A*_n_ and $C_{n}=n\cdot \frac{\pi }{4}$ represent the amplitude and phase of the movement performed by each robot joint. The followed trajectory uses cubic spline technique so as to provide not just continuity but also a zero initial velocity per link which ensures a correct physical implementation in a robot controller. This sort of trajectory is easy to follow despite the non-linearity in the robot joint angles, since both joint velocities and accelerations are constricted to small bounds depending on the amplitude and phase previously indicated (Figure 2B).

Aiming to quantitatively evaluate the movement performance in terms of accuracy, the average of the MAE per robot joint was calculated. The estimation of this measurement was monitored in each trial, thus allowing the global movement accuracy evolution during the learning process.

### Test-bed experiments

So as to provide the robot arm plant with a rich enough dynamic scenario that could fully reveal the robot arm properties, two different configurations have been tested:

- A set of different punctual heavy masses (payloads) attached to the robot end effector producing dynamic deviations when they are manipulated (light-2 kg-payload and heavy-10 kg-payload).
- An external variable force (along x, y, and z axes) which is applied to the end effector as described by Equation (8).

(8a) $$\begin{array}{l} \;\vec{F}\;=\;\left[F_{x},F_{y},F_{z}\right]\\ F_{x,y,z}\;=\;100_{x,y,z}\;\cdot \;\cos(2\;\cdot \;\pi \;\cdot \;10t\;+\;C_{x,y,z}) \end{array}$$

(8b) $$\begin{array}{l} where\;x,y,z\;=\;cartesian\;axes\\ and\;C_{x,y,z}=\left(\frac{\pi }{4},\;2\;\cdot \;\frac{\pi }{4},\;3\;\cdot \;\frac{\pi }{4}\right) \end{array}$$

This external force has 10 times the period of the trajectory movement and is in the same range than the needed torque values to operate the robot plant. This new scenario demands not just fast adjustment in agonist-antagonist pair of muscles (the error in each joint goes from positive to negative along the trajectory execution) but also a fast control due to the quick changeable working point that the robot-arm undergoes. This set up including external forces is often used to evaluate potential roles of the cerebellum in control tasks (Witney et al., 2000; Howard et al., 2010).

## Results

In these simulations, the cerebellar model delivered to a robotic arm the corrective actions needed to compensate for dynamic deviations produced by manipulating heavy objects (Garrido et al., 2013a). Simulations were designed to evaluate whether and how specific cerebellar architectures of the model (Figure 1) could generate fast convergence and stable outputs in the initial learning stages without the need for traditional feedback controllers widely used in robotic literature (Kawato and Gomi, 1992; Stroeve, 1997; Desmurget and Grafton, 2000; Kalveram et al., 2005). We tested the hypothesis that such fast convergence could be achieved by implementing the IO-DCN connection and by endowing this latter with plasticity, thereby generating an *internal adaptable feed-back loop*. Movies of learning simulations during manipulation of a 2-kg load are shown in the Supplemental Material.

### Distributed plasticity determines learning generalization

In order to identify the impact of the different forms of plasticity, the network was sequentially added with multiple adaptive mechanisms. We have previously shown that plasticity at PF-PC synapses was not sufficient to ensure a proper adaptive manipulation of objects with different weight (Garrido et al., 2013a). By changing payload from the initial setting, Purkinje cells were easily saturated preventing them from generating appropriate corrective torques. This limitation was overcome by implementing MF-DCN and PC-DCN plasticity, thus allowing PC activity to remain within its optimal frequency range independently from the manipulated mass.

**Figure 3:**
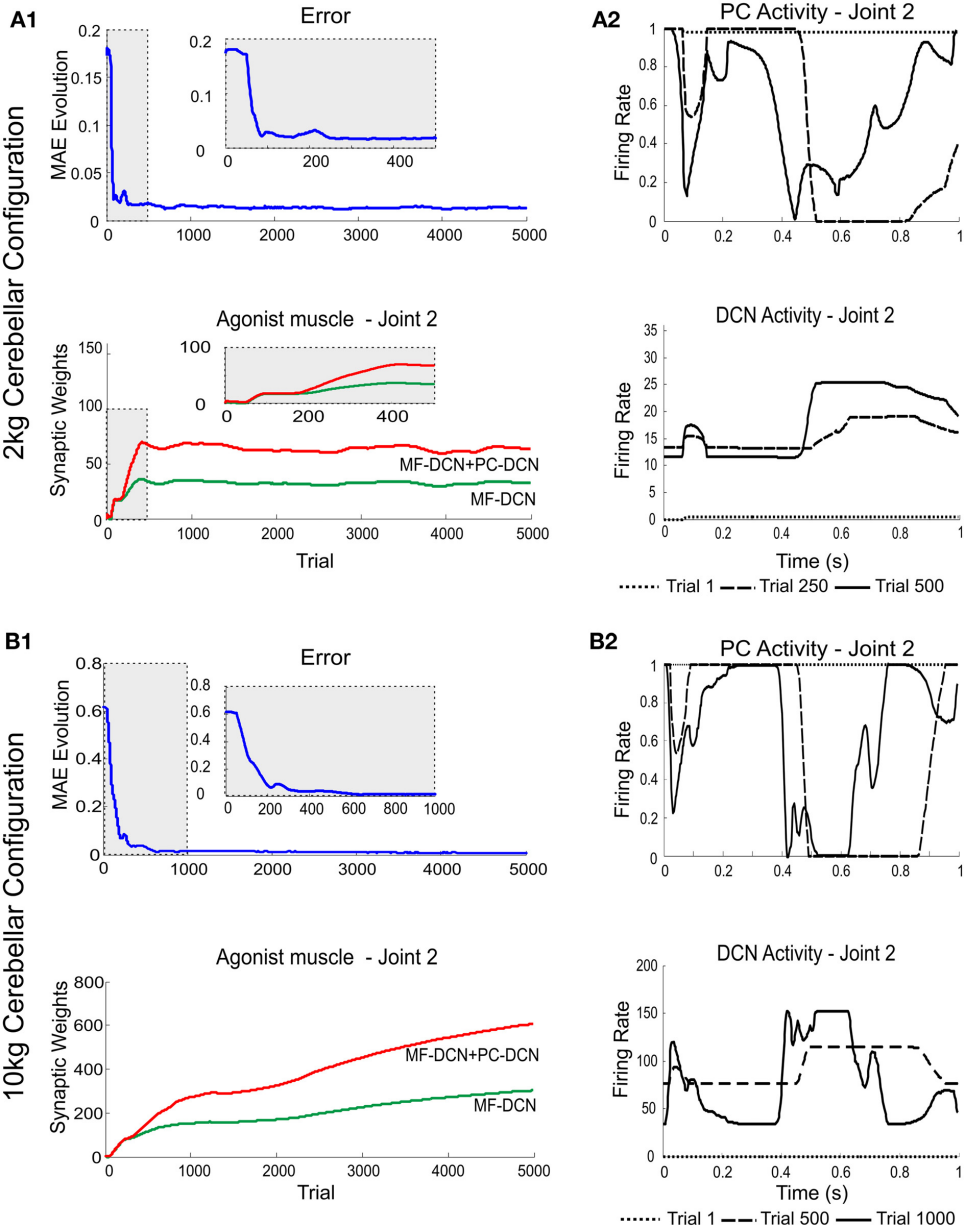
**Learning generalization by means of distributed plasticity**. The system gain (external to the cerebellum) was properly set to manipulate accurately the robot-arm without any object (no external payload). Since the manipulated mass (payload) was not expected, the existing plasticity mechanisms at MF-DCN and PC-DCN had to adjust the cerebellar output to cope with this mass (2 kg/10 kg mass configuration). **(A1)** Performance and learning when manipulating 2 kg mass. Evolution of the average MAE of the three robot joints during the learning process, 5000 trials. In the initial learning trials (zoom in) the MAE averaged value was about 10 times greater than the obtained MAE average value at the end of the learning process. MF-DCN and PC-DCN adjustments took about 500 iterations to be set, meanwhile the cerebellar system was working in open-loop and no action control was appropriately delivered. Plasticity occurred at PF-PC, MF-DCN, and PC-DCN synapses. The evolution of synaptic weights at MF-DCN, PC-DCN connections related to join 2 agonist muscle is also shown. For the sake of clarity only the behavior of this second joint is shown, however similar results were found throughout the learning process in both joints 1 and 3. MF-DCN and PC-DCN synaptic weight stabilization was obtained from the 500th trial. **(A2)** Normalized PC Firing rate (top) and DCN firing rate (bottom) during different trials taken from the initial stages of the learning process: trial 1, trial 250, and trial 500. MF-DCN and PC-DCN synaptic weight adjustments allowed the PC/DCN firing rate to operate in a proper range. **(B1)** Performance and learning when manipulating 10 kg mass. Evolution of the average MAE of the three robot joints during the learning process, 5000 trials. In the initial learning trials (zoom in) the MAE averaged value was, roughly speaking, more than 30 times greater than the obtained MAE average value at the end of the learning process. MF-DCN and PC-DCN adjustments took about 1000 iterations to settle down, meanwhile the cerebellar system was working in open-loop, and hence no action control was appropriately delivered. Plasticity occurred at PF-PC, MF-DCN, and PC-DCN synapses. The evolution of synaptic weights at MF-DCN, PC-DCN connections related to join 2 agonist muscle is also shown. For the sake of clarity only the behavior of this second joint is shown, however similar results were found throughout the learning process in both joints 1 and 3. MF-DCN and PC-DCN synaptic weight stabilization was obtained from the 3000th trial. **(B2)** Normalized PC firing rate (top) and DCN firing rate (bottom) during different trials taken from the initial stages of the learning process: trial 1, trial 500, and trial 1000. MF-DCN and PC-DCN synaptic weight adjustments allowed the PC/DCN firing rate to operate in a proper range.

A first simulation was carried out to show how self-regulation of MF-DCN and PC-DCN synapses could improve convergence in the cerebellar control loop. During a manipulation task, a mass was moved along a 1 s trial trajectory repeated 5000 times (Figure 2). The learning process occurred when a 2-kg payload was manipulated starting from a 0-kg initial configuration. After DCN synaptic weight adaptation (Figure 3A1), the cerebellum was able to deliver proper corrective torques reducing the error of the robot-arm movement close to 0 (Figure 3A1). Once synaptic weights were stabilized, both PC and DCN neurons exploited their whole firing range (Figure 3A2) allowing the cerebellum to operate near its optimal performance. This system could effectively generalize toward the subsequent application of a 10-Kg payload (Figures 3B1,B2).

This correction was precise but learning was slow, as it took about 500/1000 (2 kg/10 kg configuration) repetitions (Figures 3A1,A2,B1,B2). Thus, the precise commands could not be properly delivered by the control system until the cerebellar learning process was complete. Throughout the adaptation period, the cerebellum operated in open-loop (this was well evident during the first learning stages, where the cerebellum was hardly starting to adapt). Therefore, an effective feed-back system was required to accelerate learning.

### The IO-DCN connection accelerates learning with fixed IO-DCN weights

In order to evaluate the effectiveness of the IO-DCN connection in controlling adaptation during the initial learning stages, the IO-DCN synaptic weights were pre-calculated to handle different masses with the same values (light mass: 2 kg-payload; heavy mass: 10 kg-payload). Then the IO-DCN synaptic weights were kept fixed and the MF-DCN and PC-DCN weights were allowed to self-adapt during the learning process composed of 1 s trial trajectories repeated 5000 times using either light or heavy payloads.

**Figure 4:**
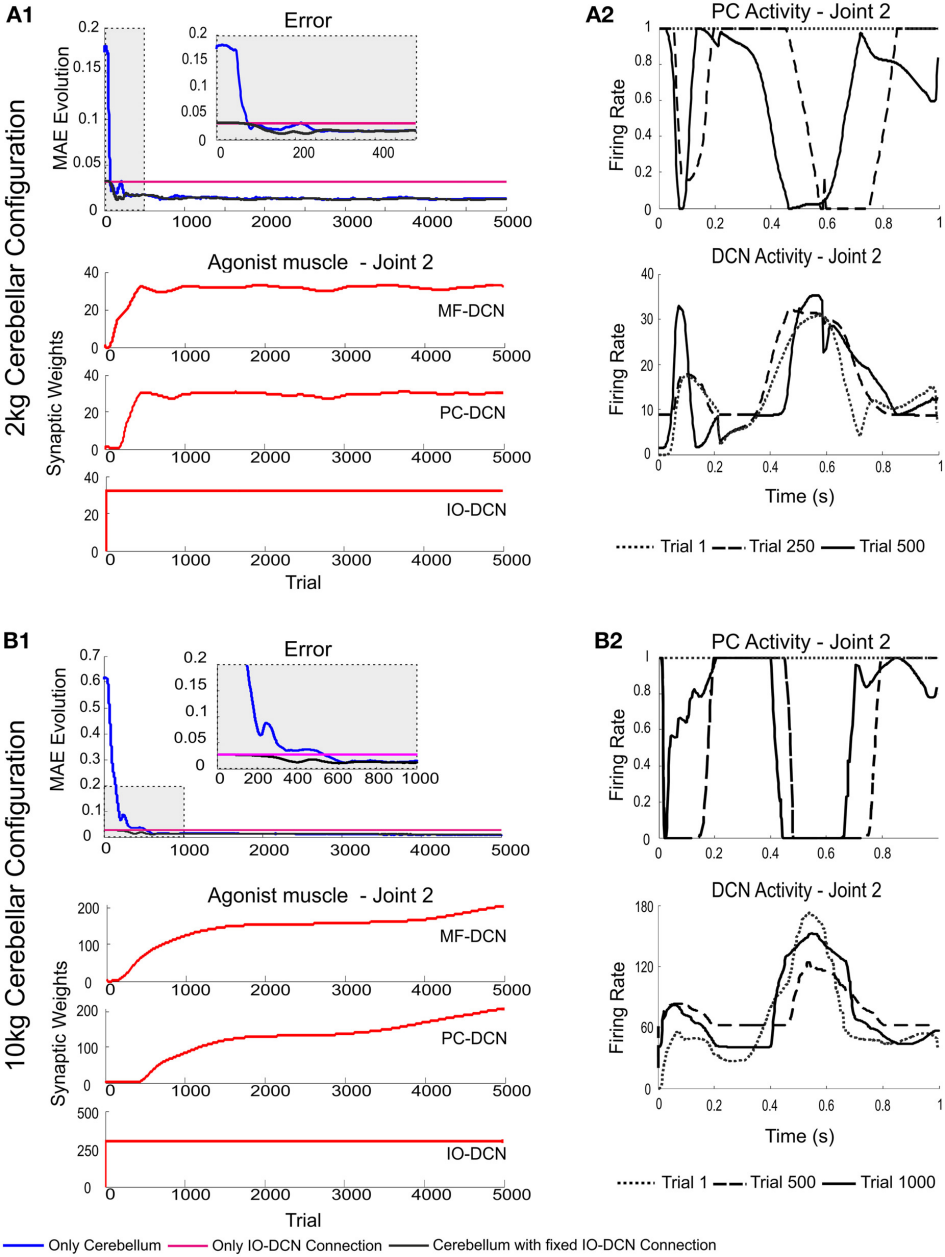
**Weight evolution in the cerebellar model manipulating different payloads with IO-DCN connection operating with multiple plasticity mechanisms**. Simulations were performed using plasticity mechanisms at PF-PC, MF-DCN, and PC-DCN synapses using a custom-configured IO-DCN connection for manipulating 2 and 10 kg external payloads during 5000 trials. The initial cerebellar system gain was properly set to operate with no payload. **(A1,B1)** Evolution of the average MAEs of the three robot joints during the learning process for 2 and 10 kg payloads respectively with/without IO-DCN fixed synaptic weights plus cerebellum or with just the IO-DCN connection. Note that the configuration without IO-DCN connection adjusted the DCN gain after approximately 500/1000 (2 kg/10 kg configuration) trials on average. From the first trial to the 500th/1000th (2 kg/10 kg configuration) the cerebellar system worked almost in open loop, no remarkable corrective action was applied by the cerebellar adapting system. The configurations with or just IO-DCN connection were capable of supplying a proper adjustment from the beginning of the learning process. **(A2,B2)** Evolution of synaptic weights at IO-DCN, MF-DCN, and PC-DCN connections related to join 2 agonist muscle. For the sake of clarity only the behavior of this second joint is shown, however similar results were found along the learning process in both joints 1 and 3. MF-DCN and PC-DCN weights stabilized in about 500/3000 trials (2 kg/10 kg configuration) at different convergence speeds. This slow convergence was the consequence of the existing inter-dependence between the PC-DCN learning and the DCN activity which also depended on both MF-DCN and PC-DCN adaptation. IO-DCN connection supplied cerebellar control action whilst MF-DCN and PC-DCN synaptic weights were not stable yet.

Using pre-calculated synaptic weights allowed the IO-DCN connection to operate over the whole learning process providing a rough control facilitating the DCN to operate in its pseudo-optimal firing rate from the very beginning (Figures 4A2,B2). Pre-calculated IO-DCN connections, even though with fixed weight values, contributed to error reduction especially in the first 200/600 trails (2 kg/10 kg configuration). The contribution of IO-DCN connections was enough to enable a corrective control that improved the precision of the manipulation tasks (Figures 4A1,B1). Plasticity at MF-DCN and PC-DCN synapses contributed to further increase the precision of the manipulation task within about 500/1000 trials (Figures 4A1,B1). This slow convergence was due to the inter-dependence of PC-DCN learning on DCN activity which, in turn, depended on both MF-DCN and PC-DCN adaptation (see Methods). Actually, the fact that adaptation of MF-DCN and PC-DCN weights was slow (Figures 4A1,B1) made IO-DCN connection the only DCN afferent synapse able to control the manipulation task during the first trials. Thus, the IO-DCN connection was crucial for facilitating the cerebellar circuit to approximate the ideal corrective torques from the very beginning of learning.

### Plasticity at IO-DCN synapses contribute to the distributed learning process enhancing motor performance

In order to further evaluate its impact on the initial learning stage, the IO-DCN connection was made self-adaptive. We evaluated how these adaptive IO-DCN synaptic weights optimize payload manipulation. The IO-DCN weights were allowed to self-adapt during a learning process composed of 1 s trial trajectories repeated 5000 times using either light or heavy payloads.

**Figure 5:**
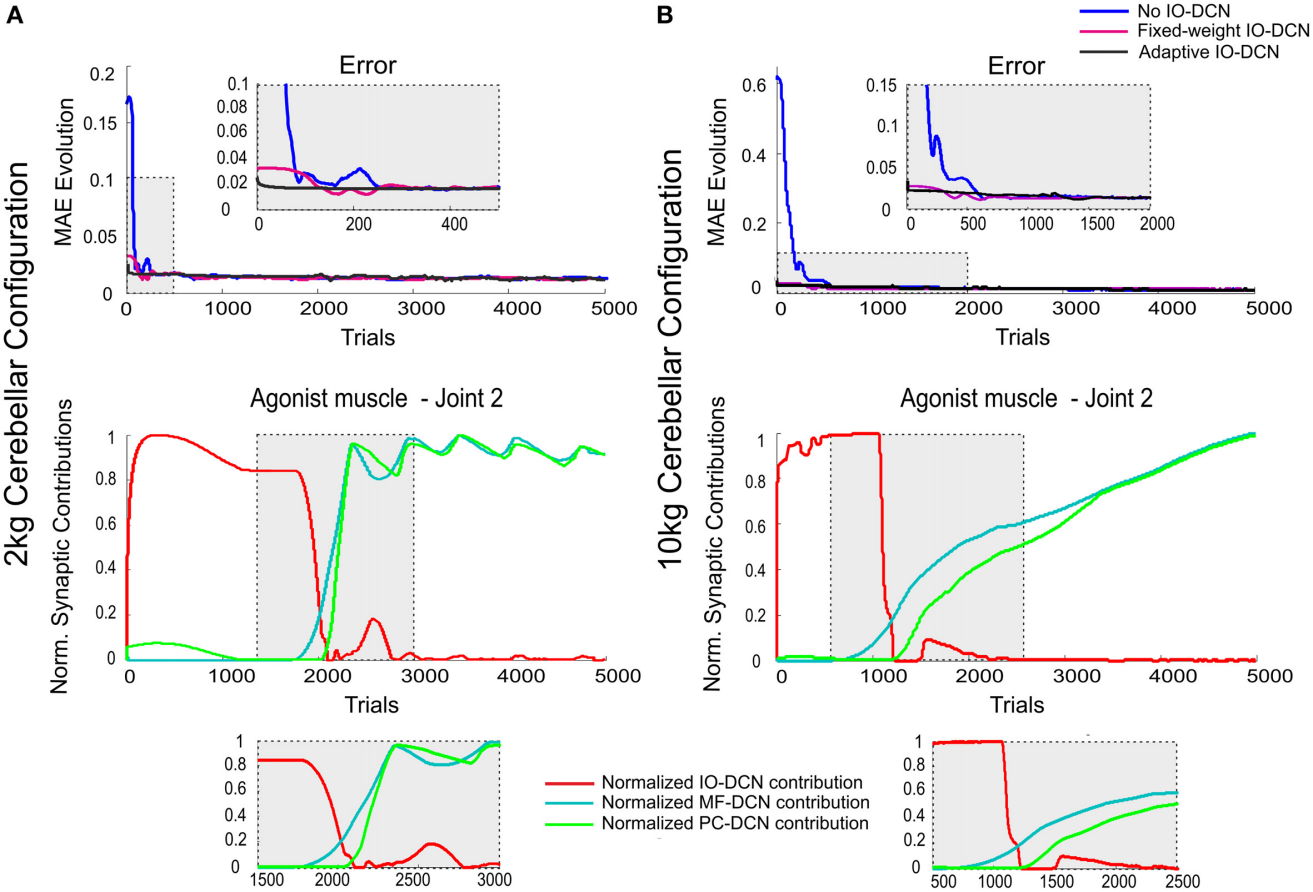
**Normalized synaptic contribution of each DCN afferent throughout the learning process evolution using a self-adaptable IO-DCN connection**. Simulations were performed using plasticity mechanisms at PF-PC, MF-DCN, and PC-DCN synapses using a self-adaptive plasticity mechanism at IO-DCN connection for manipulating 2 and 10 kg external payloads during 5000 trials. The evolution of the average MAEs of the three robot joints during the learning process for 2 kg **(A)** and 10 kg payloads **(B)** with a cerebellum equipped with IO-DCN connection with/without self-adaptive synaptic weights is presented. The initial cerebellar system gain was properly set to operate with no payload. Since the manipulated masses were unexpected, the existing plasticity mechanisms at MF-DCN and PC-DCN adjusted the cerebellar output to cope with these masses. At initial learning stages, the cerebellar model presenting an adjustable IO-DCN connection provided a more accurate corrective action to properly perform the manipulation task. The distributed adaptation of IO-DCN, MF-DCN, and PC-DCN synaptic strengths when using 2 kg **(A)** and 10 kg payloads **(B)** related to join 2 agonist muscle is also presented. For the sake of clarity only the behavior of this second joint is shown, however similar results were found throughout the learning process in both joints 1 and 3. The self-adjustable IO-DCN connection was capable of supplying a proper adjustment from almost the beginning of the learning process. The control action of this connection was relevant only in early learning stages; once the learning process settled down, the IO control action became negligible (see zoom-in of normalized synaptic weight evolution plots).

During the initial learning stage, the IO-DCN corrective action dominated (Figures 5A,B, bottom). Then this corrective action gradually decreased, whilst that provided by MF-DCN and PC-DCN connections gradually increased (Figures 5A,B, bottom). The transition between these two control phases was regulated by PCs, whose activity was maintained within a narrow frequency range through the adjustment of MF-DCN and PC-DCN connections reverberated through the control loop.

In this configuration, all the learning sites were working complementing each other, generating an effective distributed learning network (Figure 5). The addition of self-adaptive IO-DCN connections was crucial not only to accelerate delivery of corrective torques stabilizing motor outputs during initial learning stage, but also to facilitate the balanced learning at MF-DCN and PC-DCN connections.

### The impact of the “modulated term” at self-adaptive IO-DCN connections

In simulations shown in Figures 3–5, the IO-DCN correction proved critical to provide effective control over the initial learning stage. This control was regulated by the *modulating term* (*MTP/MTD*) (see Equation 6). The impact of the modulating term was assessed on manipulation of a 2 kg payload. In the meantime, MF-DCN and PC-DCN weights were also allowed to self-adapt during the learning process composed of 1 s trial trajectories 5000 times.

**Figure 6:**
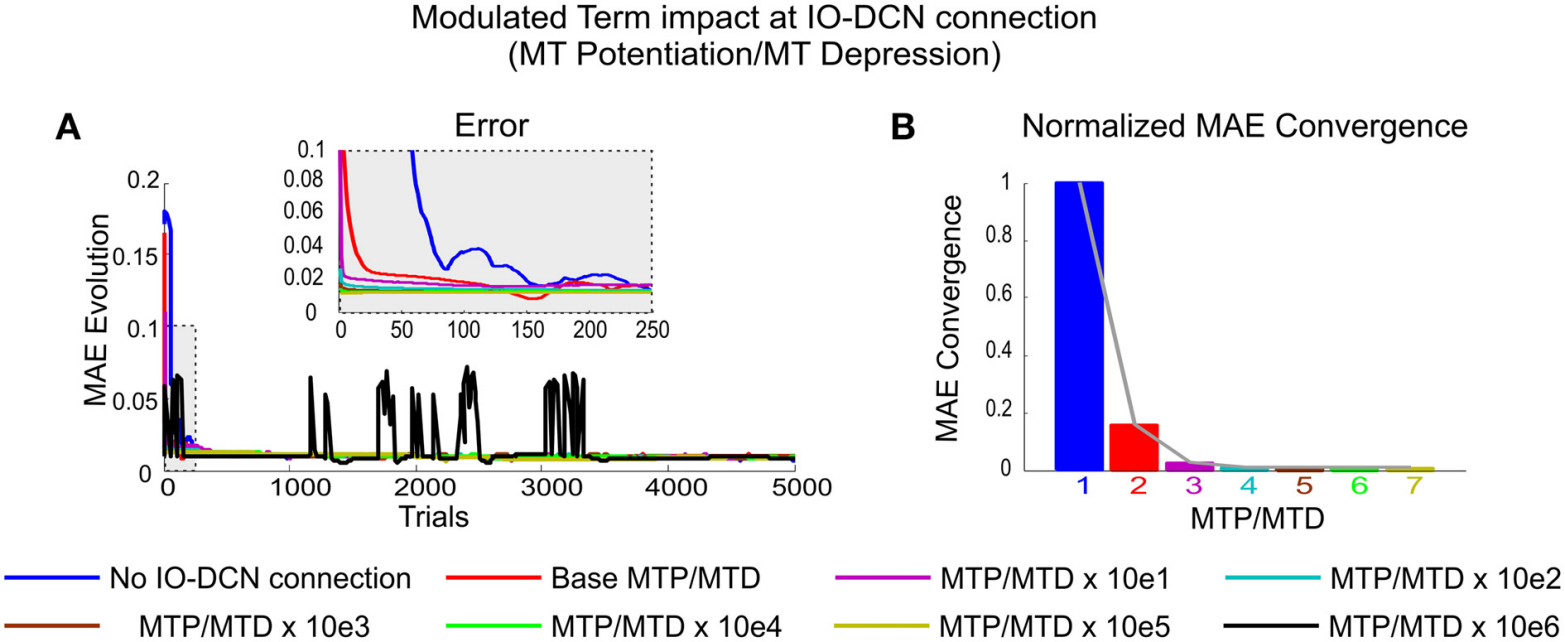
**Modulated Term impact at self-adaptive IO-DCN connection**. Simulations were performed using plasticity mechanisms at PF-PC, MF-DCN, and PC-DCN synapses using a self-adaptive plasticity mechanism at IO-DCN connection for manipulating 2 kg external payload during 5000 trials. **(A)** Evolution of the average MAEs of the three robot joints during the learning process for a 2 kg payload with a cerebellum equipped with a self-adaptive synaptic weight IO-DCN connection. The modulating term plasticity at IO-DCN connection (see Equation 6) was ranged from base MTP/MTD values of 0.001 to 1000 respectively. The higher the values, the faster and the stabler the system converged. At values greater than 100 the system became unstable, a sort of **windup** effect appeared. The IO-DCN connection control command exceeded the physical limits of the robot-arm-system (it delivered a more corrective action at each integration step than the system could handle and needed). The IO-DCN connection control momentum was incapable of immediately responding to changes in the next-integration-step incoming error. **(B)** Normalized MAE convergences obtained during the learning process for a 2 kg payload when the modulating term plasticity at IO-DCN connection ranged from [0.001, 100].

Low MTP/MTD values (from 0.001 to 1) caused a smooth self-regulated IO-DCN action or, in other words, an IO-DCN optimal corrective action was not obtained at the very first trial (Figure 6A). High MTP/MTD values (from 1 to 1000), caused a sharp self-regulated IO-DCN action. However, MTP/MTD values higher than 10e 6 (black line plot) made the arm-robot-system unstable (Figure 6A). At this point, a *windup* effect appeared. Wind-up occurred when the IO-DCN connection control command exceeded the physical limits of the arm-robot-system (i.e., the corrective actions delivered at each integration step were more than those the arm-robot-system could handle).

In other words, the IO-DCN connection control momentum was incapable of responding instantaneously to changes in the next-integration-step-incoming error. In this case, the rate of IO-DCN synaptic weight evolution was faster than the error-speed of the robot-arm-system. Thus, the IO-DCN corrective action control exceeded by far the optimal control value but it kept on growing in the very same integration step. When the next-integration-step-incoming error reached the cerebellar system, the sign of the error was then reversed, thus causing the IO-DCN control action to start “winding” down. (Figure 6A, black line plot).

Therefore, beyond the unstable point, as predicted by the windup theory, the output of the IO-DCN corrective action was decoupled from the optimal IO-DCN synaptic weight. It demanded a significant amount of time so as to recover the proper optimal range, thus causing certain lags (overshooting transients) in the cerebellar response as shown in Figure 6A, black line plot. As in any other sort of windup effect, this process may occur repeatedly or eventually converge depending on global cerebellar control gain (IO-DCN, MF-DCN, and PC-DCN synaptic weigh balance) and the robot-arm-system response.

The effect of the modulated term MTP/MTD is shown in Figure 6B. In all cerebellar configurations equipped with an IO-DCN connection the number of samples needed to reach the final average error decreased exponentially with increasing MTP/MTD values. When reaching a certain MTP/MTD value, the cerebellar system was able to deliver the appropriate adjustment of DCN synaptic weights from the very beginning of the learning process. MTP/MTD values beyond this limit caused neither a faster convergence nor a better accuracy. The exponential nature of the convergence speed meant that, when reaching a certain MTP/MTD value, the cerebellar performance was stabilized. In conclusion, the MTP/MTD regulated the speed at which the contributions of IO-DCN and MF-DCN/PC-DCN connections progressively combined facilitating an accurate and stable learning process and providing the cerebellar system with the capability of self-adaptation from the initial learning stage.

### Improved control of perturbing forces

To demonstrate whether the IO-DCN connection contributed to cerebellar control in more demanding operative scenarios, the system was exposed to an external force field. The force field was made up of an external set of variables and periodical forces (along x, y, and z axes) applied to the robot arm end-effector (see Equation 8). In these simulations, all adaptation sites at PF-PC, MF-DCN, PC-DCN, and IO-DCN synapses were enabled. The learning process occurred over 1 s trial trajectories repeated 5000 times and the MTP/MTD value was set to 10.

**Figure 7:**
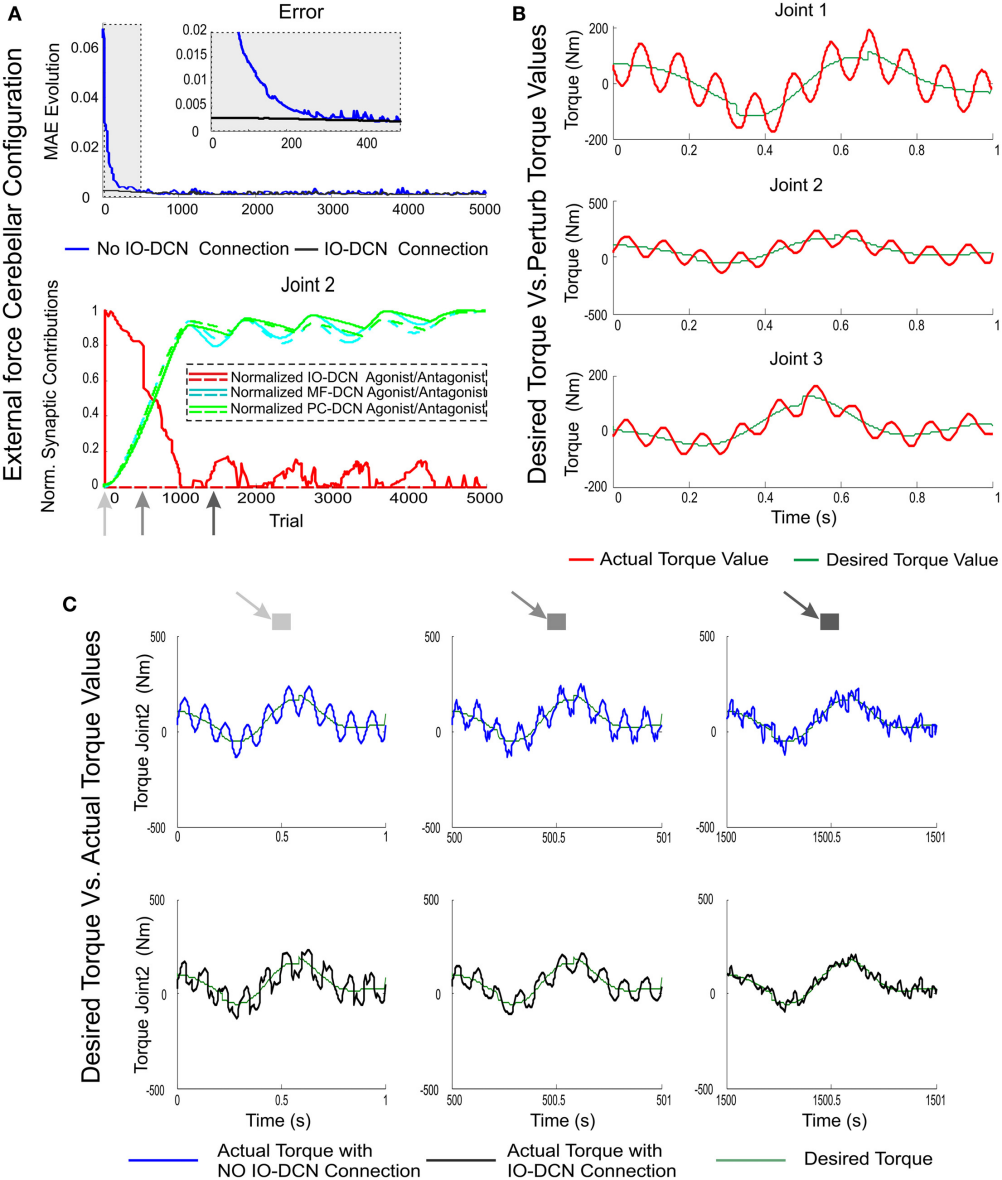
**MAE and synaptic strength evolution during the learning process at IO-DCN, MF-DCN, and PC-DCN synapses when an external variable force is applied to the end effector**. Simulations were performed using plasticity mechanisms at PF-PC, MF-DCN, and PC-DCN synapses accompanied with a self-adaptive plasticity mechanism at IO-DCN connection under an external variable force field during 5000 trials. **(A)** Illustrates the mean absolute error evolution of the three robot joints during the learning process throughout 5000 trials accompanied with a zoom-in of the first 500 trials with/without the self-adaptive plasticity mechanism at IO-DCN synapses. Plot **(A)** also illustrates the distributed adaptation of the normalized synaptic strength evolution at MF-DCN, PC-DCN, and IO-DCN connections (for the sake of clarity just second agonist and antagonist paired joint muscle have been represented). The contribution of the IO-DCN (red line) connection from the 1st trial was maximal; but the agonist/antagonist balance was not properly settled down. Agonist IO-DCN connection supplied a position-error-base- control action, thus facilitating the proper PF-PC firing range operation. The combined contribution of MF-DCN/PC-DCN connections (green and blue lines) became strong enough from 500th trial to keep the system under control allowing a fine-tuning of agonist/antagonist balance whilst the IO-DCN contribution was progressively self-neglected. **(B)** Perturbing torque values resulting from force field action compared to desired torque values needed to perform the eight-like trajectory **(C)** Torque value evolution during the learning process for the second joint with/without the self-adaptive plasticity mechanism at IO-DCN synapses. Three time stamps were shown: 1, 500, and 1500 trial. IO-DCN contribution was responsible for correcting the initial torque output in a rough manner (first trial). With the passage of time the fine agonist/antagonist balance at MF-DCN/PC-DCN connections allowed the arm-robot-system to compensate torque deviations due to force field action.

The actual torque values operating the robot-arm-end effector under a variable periodic force field are shown in Figure 7B. This force field made the torque values exceed the magnitude of ideal ones. To accurately perform the eight-shape trajectory, the cerebellum was committed to compensate the existing difference between them. As expected, the manipulation problem increased its complexity compared to the unperturbed task; throughout a learning trial, both torque gradient and torque value in each robot-arm-joint were continuously changing (sign and magnitude), thus making the agonist-antagonist synaptic weight adaptation become crucial during the learning process. The mismatch between actual joint position and ideal ones was compensated thanks to the IO-DCN connection (Figure 7A). An initial rough control action was delivered, allowing the cerebellar system to provide a corrective torque in ameliorating manipulation (Figure 7C).

The contribution of the IO-DCN (Figure 7A) connection was maximal in the initial learning stage. However, the agonist/antagonist balance could not be properly achieved initially. The IO-DCN connection initially supplied a position error-based control that made PF-PC synaptic activity to operate in its optimal range. The position error was early compensated (Figure 7A) whilst the agonist/antagonist balance required (Figure 7A) the combined MF-DCN/PC-DCN adaptation (Figure 7C, green and blue lines). Whilst time was passing, the IO-DCN contribution was progressively substituted by the distributed learning at MF-DCN/PC-DCN synapses, and the contribution to the DCN output at final learning stages was completely supplied by these latter connections. During the final learning stages, the learning process stabilized (Figure 7A, MAE plateau phase), thus inferring and storing in DCN synapses the control action needed to compensate the external variable force (Figure 7C).

## Discussion

By using closed-loop robotic simulations, this paper reveals the internal dynamics of long-term plasticity and neuronal firing in a cerebellar network model incorporated into a system control platform. The cerebellar network exploits the IO-DCN connection to implement an internal feed-back loop and embeds multiple forms of synaptic plasticity (Garrido et al., 2013a). The main observation is that plasticity at the IO-DCN connection accelerates convergence of learning by 1–2 orders of magnitude. In this way learning approaches the speed demonstrated by behaviorally relevant tasks such as eye-blink classical conditioning (Smith et al., 2006) and force-field adaptation (Shadmehr et al., 2010).

IO-DCN plasticity was modeled as a Hebbian learning mechanism and was added to MF-DCN and PC-DCN plasticity, so that three different forms of plasticity impinged on the same DCN neurons. Significantly, IO-DCN plasticity caused a learning acceleration that synergized with the error-detection and generalization properties conferred by the other DCN synapses. This model therefore suggests that a putative form of IO-DCN plasticity may play a critical role in controlling DCN activity and determining the cerebellar output, mainly at improving the learning speed and maintaining certain control stability from the very initial stages, which is also important to avoid potential damage caused by unstable behaviors.

### The impact of IO-DCN plasticity on cerebellar learning

These simulations revealed three remarkable functional aspects involving the IO-DCN connection.

1. Firstly, the feedback control loop through the IO-DCN connection ensured stability during the initial phase of the learning process.
2. Secondly, the distributed learning process through different pathways ensured a relatively fast synaptic weight strength adjustment by using the PF-PC plasticity mechanism and the subsequent slow adaptation of the excitation and inhibition levels by means of the MF-DCN and PC-DCN synaptic plasticity mechanisms. These mechanisms also helped to keep the PF-PC synaptic weights working within their optimal range.
3. Thirdly, regulation of DCN neuron discharge was dynamic in that plasticity at its synapses evolved over time.

Thus, plasticity served to regulate DCN neuron excitability, and this regulation required the dynamic intervention of the whole cerebellar network.

### Insights on cerebellar learning

There is no agreement about the type of information conveyed by the climbing fibers into the cerebellum or about their potential role. The Marr-Albus motor learning theory maintains that climbing fibers carry either an error signal related to directional information (Kawato and Gomi, 1992) or a binary teaching signal (Houk and Barto, 1992; Bazzigaluppi et al., 2012; De Gruijl et al., 2012). Conversely, considering the periodic nature of climbing fiber activity, others (Llinas and Welsh, 1993) maintain that IO activity is related with the timing of movement. However, investigations in which this periodicity was not observed (Keating and Thach, 1995) suggested that the climbing fiber activity was correlated with the onset of movements. The controversy extends to IO functional properties, which are not yet univocally defined (De Zeeuw et al., 1998; Bengtsson and Hesslow, 2006; Welberg, 2009). Finally, different cerebellar plasticity mechanisms have recently been observed in the cerebellum suggesting that motor learning may not be exclusively related to climbing fiber activity (Hansel et al., 2001; Evans, 2007; Ohtsuki et al., 2009; D'Angelo, 2010). In the present model, the climbing fibers provide a teaching signal driving long-term synaptic plasticity both at the IO-PC and IO-DCN connections.

The present simulations suggest that cerebellar gain control can be adjusted through MF-DCN and PC-DCN synaptic plasticity working in equilibrium with IO-DCN plasticity. The homeostatic mechanisms that allow this balance are implemented by using different learning laws which drive the cerebellar model to improve its learning accuracy. IO-DCN connections ensure stable outputs in the early learning stages, when the strength of MF-DCN and PC-DCN connections is not set yet through the learning process. When the strength of the synaptic weights of MF-DCN and PC-DCN connections begins to stabilize, the synaptic strength of the IO-DCN connection diminishes. Therefore, at the end of the learning process, the effect of the IO-DCN connection in determining the cerebellar output is negligible. Nonetheless, the IO-DCN connection remains ready to act when new unexpected patterns have to be learnt. In addition, a proper synaptic weight adjustment at DCN synapses allows the PFs to operate over their complete frequency range, enhancing the precision of the cerebellar output.

To sum up, the IO-DCN pathway allowed a global feedback error reduction facilitating early and fast corrections. The MF-PF-PC-DCN system operated by achieving more accurate corrections in the long term but it required slow learning (Luque et al., 2011b).

### Biological realism and model limits

Here we have used a set of assumptions in order to generate a model which is biologically realistic but also mathematically tractable. The limits imposed by such a modeling approach, which were previously discussed in Garrido et al. (2013a), are considered here under the light of the improvements conferred to network functionality by the introduction of IO-DCN plasticity.

1. Two main assumptions are that PCs operate as state-error correlators and that the granular layer acts as a state generator (states that are unambiguous throughout the trajectory, they may be generated in relation with the movement timing or with the sensory-motor states). Since the exact function of these structures is not fully resolved, an assessment of the assumption may come from a reverse engineering approach. Electrophysiological analysis has revealed complex properties in cerebellar neurons and plasticity mechanisms (D'Angelo and De Zeeuw, 2009; De Zeeuw et al., 2011). Here, plasticity mechanisms are implemented, neglecting details on signaling cascades and the neurons are not spiking. It remains to be established whether a biologically precise representation of plasticity mechanisms and spike generation (e.g., Solinas et al., 2010) could substantially modify the core conclusion of this model.
2. The feed-back signals required to correct the actual movement, in addition to be conveyed to the cerebellum through the internal feedback passing through the IO-DCN connection, also arrive through sensory afferents (MFs) and the motor cortex (Kawato et al., 1987; Siciliano and Khatib, 2008). Moreover, the teaching signal is probably not only conveyed through the IO but also through the granular layer (Krichmar et al., 1997; Kistler and Leo Van Hemmen, 1999; Anastasio, 2001; Rothganger and Anastasio, 2009). The introduction of these further elements is expected to increase the level of flexibility and efficiency in motor control and learning.
3. We did not include the basal ganglia in our system controller. Recent evidence has shown the existence of di-synaptic pathways connecting the cerebellum with the basal ganglia (Bostan et al., 2013). Both cerebellum (Swain et al., 2011) and basal ganglia (Bellebaum et al., 2008) have been suggested to contribute to reward-related learning tasks, but how these subsystems interact and reciprocally improve their operations remains an open issue.
4. We have included in the model what, as far as we know, is the most complex set of plasticity mechanisms ever considered for the cerebellar network. However, there are multiple sub-forms of plasticity in PC and GC connections as well as in PC and GC intrinsic excitability (Hansel et al., 2001; Gao et al., 2012; D'Angelo, 2014). The integration of the present model into a spike-timing computational scheme including multiple PC plasticity mechanisms and MF-GC plasticity rule remains a future challenge.
5. Finally, and most importantly, it is worth mentioning that there is neither a clear understanding of the information processing nor a fully-detailed description of the DCN. Although the implications that the IO-DCN excitatory pathway remain yet to be demonstrated (Baumel et al., 2009; Uusisaari and De Schutter, 2011), there are biological indications pointing to the existence of climbing fiber collaterals contacting DCN (Uusisaari and Knöpfel, 2011) as well as MF collaterals. In detail, there is physiological evidence in mice indicating that both MF and CF collaterals are contacting the very same sub region (neuron group) within the DCN (Uusisaari and Knöpfel, 2011). Given the fact that the presence of specific sites and signs of plasticity at DCN is an open issue together with the MF/CF collaterals contacting the same DCN neuron group which may lead to a plausible scenario where not only MF-DCN collaterals undergo some form of plasticity.
  Indeed, the lack of biological evidence in terms of IO-DCN plasticity makes the presented working hypothesis remain speculative awaiting new physiological experiments that could provide evidence to refute/validate this.

### Theoretical implications

This model lies halfway between a classical black-box model and a realistic biological model. A non-trivial consequence of the way the model is constructed is that of providing a prediction about the need for IO-DCN plasticity, which speeds up learning. Moreover, this model could be compared to prototypical cases elaborated for dynamic neural networks (Spitzer, 2000; Hoellinger et al., 2013). In these networks, learning of complex tasks is better accomplished when the number of hidden neurons increases, as they form complex categories that are needed to interpret the multi-parametric input space. This also introduced multiple time-constants. As a whole, the greater the number of plasticity sites involved, the more extended and diversified the learning properties approaching the complexity observed in real life. This model thus suggests that multiple distributed learning mechanisms provide the key for explaining the complex properties of biological learning and prompts the search for yet unknown forms of synaptic plasticity in the cerebellar network.

## Conclusions

Whilst it has been claimed that *“the cerebellum should be regarded as a control machine rather than a learning machine”* (Rokni et al., 2008), a different view states that *“the cerebellum certainly acts as a control machine, but on top of that the cerebellum (particularly the cerebellar cortex) provides a giant switchboard for associative learning”* (Ohtsuki et al., 2009). Our model, though the intervention of plasticity at the IO-DCN connection, establishes a connection between these apparently divergent opinions. Whilst distributed synaptic plasticity mechanisms may play an important role in learning consolidation, the IO-DCN connection may act as *an embedded feedback controller* ensuring stability in the first stages of the learning process. These results also imply that degradation or malfunctioning of the IO would affect fast adaptation in the early stages of the learning of a control task. The predicted role of the IO-DCN pathway for fast cerebellar adaptation could be tested by using genetically modified animals (for review see Ito, 2013).

### Conflict of interest statement

The authors declare that the research was conducted in the absence of any commercial or financial relationships that could be construed as a potential conflict of interest.

## Abbreviations

PF: parallel fiber
MF: mossy fiber
CF: climbing fiber
GC: granule cell
GoC: Golgi cell
PC: Purkinje cell
DCN: deep cerebellar nuclei
IO: inferior olive
MLI: molecular layer interneuron
MAE: mean average error.
