# Supplementary material for: Fast convergence of learning requires plasticity between inferior olive and deep cerebellar nuclei in a manipulation task: a closed-loop robotic simulation
Source: Front Comput Neurosci. 2014 Aug 15;8:97. doi: 10.3389/fncom.2014.00097 (PMC4133770; doi:10.3389/fncom.2014.00097)
Supplement: Supplementary file 1 [file Presentation1.ZIP › Movie legends.pdf.docx]

**Movie legends**

**Movie S1 |** Learning simulation. Joint-2-related activity and synaptic weight evolution during manipulation of a 2-kg load is plotted. Simulations were run using plasticity mechanisms at PF-PC, MF-DCN, and PC-DCN with no IO-DCN connection along 5000 trials. Only 1 of every 10 trials is shown. The movement has been recorded in real-time (each trial lasts 1 s) evidencing the difficulty of the task. (Top left), 3D view of the actual (black) and desired (red) robot end-effector trajectory in Cartesian coordinates. (Second left), actual corrective torques during the current trial for joint 1 (blue), 2 (red), and 3 (green). (Third left), evolution of the MAE. (Bottom left), evolution of normalized IO-DCN synaptic weight contribution related to joint 2 agonist (solid line) and antagonist (dotted line) muscles. (Top right), evolution of four randomly chosen PF-PC synaptic weights. (Second to fifth rows right),evolution of PC activity, DCN activity, MF-DCN, and PC-DCN synaptic weights related to joint 2 agonist (solid line) and antagonist (dotted line) muscles. (Bottom right), evolution of normalized MF-DCN, and PC-DCN synaptic weight contribution related to joint 2 agonist (solid line) and antagonist (dotted line) muscles

**Movie S2 |** Learning simulation. Joint-2-related activity and synaptic weight evolution during manipulation of a 2-kg load is plotted. Simulations were run using plasticity mechanisms at PF-PC, MF-DCN, and PC-DCN with a manually adjusted IO-DCN connection along 5000 trials. Only 1 of every 10 trials is shown. The movement has been recorded in real-time (each trial lasts 1 s) evidencing the difficulty of the task. (Top left) 3D view of the actual (black) and desired (red) robot end-effector trajectory in Cartesian coordinates. (Second left), actual corrective torques during the current trial for joint 1 (blue), 2 (red), and 3 (green). (Third left), evolution of the MAE. (Bottom left) evolution of normalized IO-DCN synaptic weight contribution related to joint 2 agonist (solid line) and antagonist (dotted line) muscles. (Top right), evolution of four randomly chosen PF-PC synaptic weights. (Second to fifth rows right), evolution of PC activity, DCN activity, MF-DCN, and PC-DCN synaptic weights related to joint 2 agonist (solid line) and antagonist (dotted line) muscles. (Bottom right), evolution of normalized MF-DCN, and PC-DCN synaptic weight contribution related to joint 2 agonist (solid line) and antagonist (dotted line) muscles**.** As observed from the previous film, MF-DCN and PC-DCN weights stabilized in about 500 trials at different convergence speeds. This slow convergence was the consequence of the existing inter-dependence between the PC-DCN learning and the DCN activity which also depended on both MF-DCN and PC-DCN adaptation. IO-DCN connection supplied cerebellar control action (see MAE plot) whilst MF-DCN and PC-DCN synaptic weights were not yet stable.

**Movie S3 |** Learning simulation. Joint-2-related activity and synaptic weight evolution during manipulation of a 2-kg load is plotted. Simulations were run using plasticity mechanisms at PF-PC, MF-DCN, and PC-DCN with a self-adjusted IO-DCN connection along 5000 trials. Only 1 of every 10 trials is shown. The movement has been recorded in real-time (each trial lasts 1 s) evidencing the difficulty of the task. (Top left) 3D view of the actual (black) and desired (red) robot end-effector trajectory in Cartesian coordinates. (Second left) actual corrective torques during the current trial for joint 1 (blue), 2 (red), and 3 (green). (Third left), evolution of the MAE. (Bottom left) evolution of normalized IO-DCN synaptic weight contribution related to joint 2 agonist (solid line) and antagonist (dotted line) muscles. (Top right) evolution of four randomly chosen PF-PC synaptic weights. (Second to fifth rows right),evolution of PC activity, DCN activity, MF-DCN, and PC-DCN synaptic weights related to joint 2 agonist (solid line) and antagonist (dotted line) muscles. (Bottom right), evolution of normalized MF-DCN, and PC-DCN synaptic weight contribution related to joint 2 agonist (solid line) and antagonist (dotted line) muscles**.** Similarly to that observed for movie 2 IO-DCN connection supplied cerebellar control action (see MAE plot) whilst MF-DCN and PC-DCN synaptic weights were not yet stable. However, the self-adjustable IO-DCN connection was capable of supplying a proper adjustment from almost the beginning of the learning process. The control action of this connection was relevant only in early learning stages; once the learning process settled down, the IO control action became negligible (see bottom left plot).
