# Supplementary material for: Fast convergence of learning requires plasticity between inferior olive and deep cerebellar nuclei in a manipulation task: a closed-loop robotic simulation
Source: Front Comput Neurosci. 2014 Aug 15;8:97. doi: 10.3389/fncom.2014.00097 (PMC4133770; doi:10.3389/fncom.2014.00097)
Supplement: Supplementary file 2 [file DataSheet1.PDF]

Appendix A

The inverse dynamic equation defining the LWR is given by the expression:

$$\tau = M(Q)\cdot\ddot{Q} + C(Q)\cdot\left[\dot{Q}\dot{Q}\right] - D(Q)\left[\dot{Q}^2\right] + G(Q) + F\left(Q,\dot{Q}\right) \tag{A.1}$$

where τ is the torque value vector to be applied by the robot joints. Q, $\dot{Q}$, and $\ddot{Q}$ are vectors representing the positions, velocities, and accelerations of the joints, being $\left[\dot{Q}\dot{Q}\right]$ and $\left[\dot{Q}^2\right]$ vectors defined as follows:

$$\left[\dot{Q}\dot{Q}\right] = \left[\dot{Q}_1\dot{Q}_2, \dot{Q}_1\dot{Q}_3, \ldots \dot{Q}_{n-1}\dot{Q}_n\right]^T \tag{A.2}$$

$$\left[\dot{Q}^2\right] = \left[\dot{Q}_1^2, \dot{Q}_2^2, \ldots \dot{Q}_n^2\right]^T \; where\ n = number\ of\ links \tag{A.3}$$

where $M(Q)$ is the inertia matrix (the mass matrix), $C(Q)$ is the Coriolis matrix, $D(Q)$ is the matrix of centrifugal coefficients, $G(Q)$ is the gravity action on the joints and finally $F\left(Q,\dot{Q}\right)$ is the friction term. The friction term is crucial in controlling light-weight robot arms with high-ratio gear boxes owing to the fact that there are no conventional existing methods to control these robots without a massive modeling (van der Smagt 2000). At the same time, the friction term can be differentiated in two terms; dry and viscous friction components obtaining:

$$\tau = M(Q)\cdot\ddot{Q} + C(Q)\cdot\left[\dot{Q}\dot{Q}\right] - D(Q)\left[\dot{Q}^2\right] + G(Q) + F_D\left(Q,\dot{Q}\right) \pm F_V\left(Q,\dot{Q}\right) \tag{A.4}$$

where $F_D\left(Q,\dot{Q}\right)$ and $F_V\left(Q,\dot{Q}\right)$ are the modeled dry/viscous friction matrices. The first four terms of the Eq.A.4 mainly include the inherent robot dynamic parameters (inertia matrix, Coriolis/centrifugal matrix and gravitational force vector). These parameters are up to eleven per joint (inertia matrix is symmetrical:

1. Inertia tensor terms: $\left(xx_j, xy_j, xz_j, yy_j, yz_j, zz_j\right)$ where $j = \{1, 2, \ldots number\ of\ joints\}$
2. Center of Mass $\left(mx_j, my_j, mz_j\right)$ where $j = \{1, 2, \ldots number\ of\ joints\}$
3. Mass $\left(m_j\right)$ where $j = \{1, 2, \ldots number\ of\ joints\}$
4. Motor Inertia $\left(I_j\right)$ where $j = \{1, 2, \ldots number\ of\ joints\}$

These parameters are usually grouped according to these four categories in order to make the computational task easier (Khalil and Dombre 2002). For our particular LWR robot (Albu-Schäffer et al., 2007), the nominal values obtained applying parametric methods (Bona & Curatela 2005) are shown in Tables A.I, A.II and A.III.

TABLE A.I| Inertia tensor parameters $\left(kg \cdot m^2\right)$

j=joint	xx_j	xy_j	xz_j	yy_j	yz_j	zz_j
**j=1**	0.0216417	0	0	0.0214810	0.0022034	0.0049639
**j=2**	0.0244442	0	0	0.0052508	0.0036944	0.0239951
**j=3**	0.0213026	0	0	0.0210353	0.0022204	0.0046970
**j=4**	0.0231668	0	0	0.0048331	0.0034937	0.0227509
**j=5**	0.0081391	0	0	0.0075015	0.0021299	0.0030151
**j=6**	0.0033636	0	0	0.0029876	0	0.0029705
**j=7**	0.0000793	0	0	0.0000783	0	0.0001203

TABLE A.II| Center of mass (m), **Mass** (kg) **and Motor Inertia** $\left(kg \cdot m^2\right)$

j=joint	mx_j	my_j	mz_j	m_j	I_j
**j=1**	0.0	0.01698	-0.05913	2.7082	415.50e-6
**j=2**	0.0	0.11090	0.01410	2.7100	415.50e-6
**j=3**	0.0	-0.01628	-0.06621	2.5374	361.60e-6
**j=4**	0.0	-0.10538	0.01525	2.5053	138.50e-6
**j=5**	0.0	0.01566	-0.12511	1.3028	54.10e-6
**j=6**	0.0	0.00283	-0.00228	1.5686	60.08e-6
**j=7**	0.0	0.0	0.06031	0.1943	60.08e-6

TABLE A.III| Friction parameter values: Dry friction $(N \cdot m)$ **and viscous friction** $\left(N \cdot m \cdot {}^{s}/_{rad}\right)$

j=joint	F_{Dj}	F_{Vj}
**j=1**	∓0.35	2.0e-3
**j=2**	∓0.35	1.69800e-3
**j=3**	∓0.35	1.66000e-3
**j=4**	∓0.35	2.40000e-3
**j=5**	∓0.35	1.80000e-3
**j=6**	∓0.35	1.20000e-3
**j=7**	∓0.35	1.20000e-3

Appendix B

TABLE B.I| Maximum LTP/LTD values to be applied at any time per learning law

Learning laws	LTP/MTP_{max}	LTD/MTD_{max}	Initial Synaptic Weight Values
$\Delta W_{PF \to PC}$	0.02	0.01	1
$\Delta W_{MF \to DCN}$	1e-4	1e-3	1
$\Delta W_{PC \to DCN}$	1e-4	1e-3	1

$\Delta W_{IO \to DCN}$	[0.001-1000]	[0.001-1000]	1
